# Traumatic lumbar visceral herniation in a young woman^☆^

**DOI:** 10.1016/j.ijscr.2013.09.010

**Published:** 2013-10-03

**Authors:** Ashley Woolbert, Emily R. Calasanz, Muhammad Nazim

**Affiliations:** a Texas Tech University Health Sciences Center School of Medicine, c/o Department of Surgery, 1400 S. Coulter, Amarillo, TX 79106, USA; b Department of Surgery, Texas Tech University Health Sciences Center School of Medicine at Amarillo, 1400 S. Coulter, Amarillo, TX 79106, USA

**Keywords:** Lumbar hernia

## Abstract

**INTRODUCTION:**

Lumbar herniation is uncommon, with traumatic etiology being rare. Traumatic lumbar hernias are usually caused by seatbelt injury in motor vehicle accidents. It is exceedingly uncommon to see lumbar hernias in an unrestrained passenger of a motor vehicle accident.

**PRESENTATION OF CASE:**

We present a case of a traumatic inferior lumbar hernia in a young woman who was an unrestrained driver of a vehicle involved in a high-speed collision, with multiple rollover and ejection. CT scans of the abdomen and pelvis suggested soft tissue injury involving muscles in the left lower posterior flank with traumatic herniation of the colon and small bowel. Emergent midline abdominal laparotomy confirmed herniation in the left lower quadrant. After abdominal closure, in the prone position, an extensive laceration over the left flank also confirmed herniation. Due to its dirty nature, the wound was irrigated, lavaged and covered with wound vacuum-assisted closure placement. The decision was made in favor of delayed elective hernia repair.

**DISCUSSION:**

Lumbar hernias are usually caused by sudden force to the abdomen, leading to increased intra-abdominal pressure. This pressure combined with areas of weakness in the superior and/or inferior triangle lead to herniation. Uncommonly, the contents of lumbar hernias can strangulate or incarcerate leading to bowel obstruction. This can often be prevented by detection with CT and laparotomy.

**CONCLUSION:**

Lumbar herniation of traumatic etiology is rare. Early detection with CT and/or exploratory laparotomy is important to avoid increases in size of the defect and bowel strangulation and incarceration.

## 1 Introduction

Herniation in the lumbar region is rare, with traumatic etiology being even less common. Traumatic lumbar hernias are usually caused by seatbelt injury in motor vehicle accidents. These hernias are often associated with other intra-abdominal injuries. We present a case of a traumatic inferior lumbar hernia in an unrestrained passenger of a motor vehicle accident. Diagnosis was made with CT scan followed by exploratory laparotomy. The hernia contained both large and small bowel. There was no incarceration or perforation of the bowel and no associated intra-abdominal injuries were found.

## 2 Presentation of case

A 34-year-old woman was brought in as a level-1 trauma to the emergency department. According to Emergency Medical Services, the patient was an unrestrained driver of a small vehicle involved in a high-speed accident with multiple rollover. The patient was ejected and landed on a barbed wire fence. The patient was intubated at the scene for airway and unstable vital signs. The patient had complete amputation of her right upper extremity as well as a deformed left upper extremity with extensive lacerations of soft tissue injury involving her entire back extending to the bilateral flanks. She was brought in in hemorrhagic shock. The patient was resuscitated according to the Advanced Trauma Life Support (ATLS) protocols along with the institution of massive transfusion protocol.

**Fig. 1:**
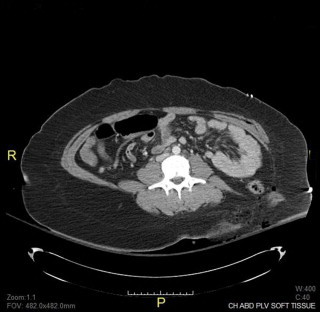
CT abdomen demonstrating small bowel and descending colon herniated through left lower abdominal wall and pelvic muscles.

**Fig. 2:**
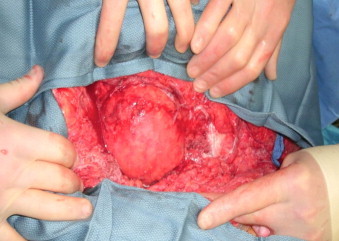
Gross intraoperative presentation of lumbar visceral hernia.

Her past medical, surgical, family and social histories were not available. After stabilization of her vitals, she underwent CT scans of the head, chest, abdomen, and pelvis along with CT angiogram of head and neck. CT results in conjunction with extremity X-rays revealed the following: small subarachnoid hemorrhage, T11 vertebral compression fracture, left-sided rib fracture, right upper extremity complete amputation, left humerus fracture, right scapular fracture, and evidence of extensive soft tissue injury involving the pelvic and the paraspinous muscles in the left lower posterior flank with traumatic herniation of the colon and small bowel (Fig. 1). She emergently underwent exploratory abdominal laparotomy which revealed no bowel injury but confirmed the herniation in the left lower quadrant. The peritoneum was found to be intact. After closing the abdomen, she was placed in a prone position revealing a complex laceration over the left flank. There was a bulge noticed in this area confirming the area of herniation (Fig. 2). A significant amount of dirt and gravel was found in this wound. The wound was irrigated and debrided and covered with wound vacuum-assisted closure placement. The decision was made to repair the hernia electively.

## 3 Discussion

The lumbar region is bordered by the external oblique laterally, the erector spinae muscles medially, the twelfth rib superiorly, and the iliac crest inferiorly. Herniation (extrusion of intraperitonal or extraperitoneal contents through a posterolateral abdominal wall defect) can occur in the superior triangle (Grynfeltt–Lesshaft's hernia), inferior triangle (Petit's hernia), or both (diffuse lumbar hernia). The superior triangle is an inverted triangle bounded by the internal oblique muscle anteriorly, the twelfth rib superiorly, and the erector spinae muscles posteriorly.^1^ The inferior triangle (Petit's) is bounded by the iliac crest inferiorly, external oblique anteriorly, and the latissimus dorsi posteriorly.^1^

Lumbar hernias may be congenital or acquired.^2^ Congenital hernias account for 20% of lumbar hernias while acquired hernias account for the other 80%.^1^ The acquired hernias can be subdivided into acquired primary lumbar hernias (spontaneous) and acquired lumbar hernias (secondary to trauma, infection, or prior surgery).^2^ The majority of lumbar hernias occur in the superior lumbar triangle.^2^ However, despite their rarity, lumbar hernias of traumatic etiology are usually in the inferior triangle.^3^ Lumbar hernias typically are caused by sudden force to the abdomen, leading to increased intra-abdominal pressure. Both the superior and inferior lumbar triangles are considered areas of weakness in the posterolateral abdominal wall.^1^ These areas of weakness combined with increased intra-abdominal pressure can produce abdominal wall defects such as lumbar hernias. Motor vehicle accidents account for about 70% of traumatic lumbar hernias, and in the majority of these cases the decelerating force of a seatbelt is the cause for the higher number of inferior triangle hernias in traumatic cases.^3^

The contents of traumatic lumbar hernias most commonly include fat, colon, and small bowel.^3^ The rates of strangulation and incarceration are low as the areas of herniation are typically wide. When strangulation or incarceration does occur, it can lead to bowel obstruction.

Traumatic lumbar hernias are rarely an isolated injury.^3^ They are often associated with other intra-abdominal injuries. Injury to the mesentery, liver, kidneys, spleen, and bowel can also occur in association with lumbar hernias. Lumbar spine injuries are also associated with traumatic lumbar hernias since both are commonly caused by seatbelt injury.^3^

The diagnosis of lumbar hernias in a trauma setting is clinically difficult because of many distracting factors such as additional organ injuries, contusions. Historically, exploratory laparotomy revealed lumbar hernias.^1^ CT scan has been found to be 98% sensitive for the diagnosis of traumatic lumbar hernias.^3^ Several undetected cases of traumatic lumbar hernias have been diagnosed with CT scans in the literature. Undetected traumatic lumbar hernias can increase in size, leading to increased long-term morbidity such as bowel incarceration and strangulation.^1^

The management of traumatic lumbar hernias has been variable. Due to the high rate of intra-abdominal injury associated with these hernias, it has been suggested that exploratory laparotomy be performed immediately.^2^ The timing of the hernia repair may vary. Some have been repaired immediately, such as cases with strangulation. Other cases have had delayed hernia repair, especially when there is a high risk for surgical infection, as was the case with our patient. Repair can be performed with mesh patches and with muscle and fascial flaps, especially for larger defects.^2^

## 4 Conclusion

Traumatic lumbar hernias are exceedingly rare with less than 300 cases described in the literature.^2^ In a hemodynamically stable patient, a CT scan may be performed prior to surgery as it is the most sensitive and specific mode of diagnosis. Repair is circumstantial depending on the surgeon's preference, the nature of the surrounding tissue, and the risk of infection. In most cases, repair should be done within a reasonable time frame to avoid an increase in size of the defect and morbidity such as bowel strangulation and incarceration.

## Conflict of interest

We have no conflicts of interest to declare.

## Funding

None.

## Ethical approval

Written informed consent was obtained from the patient for publication of this case report and accompanying images. A copy of the written consent is available for review by the Editor-in-Chief of this journal on request.

## Author contributions

Emily R. Calasanz: Contribution to writing of manuscript.

Ashley Woolbert: Contribution to manuscript preparation.

Dr. Nazim: Participation in preoperative, intraoperative, and postoperative care. Contribution to manuscript preparation.
